# Supplementary material for: Epidemiological characteristics of invasive meningococcal disease and carriage prevalence of Neisseria meningitidis in the Xinjiang Uygur Autonomous Region, China, 2004–2023: a retrospective study
Source: PeerJ. 2025 Jul 29;13:e19772. doi: 10.7717/peerj.19772 (PMC12315827; doi:10.7717/peerj.19772)
Supplement: Supplemental Information 2 [file peerj-13-19772-s002.docx]

STROBE Statement—Checklist of items that should be included in reports of ***cross-sectional studies* Item**

**No Recommendation Page No**

| **Title and abstract** | 1 | Epidemiological Characteristics of invasive meningococcal disease and Carriage Prevalence of Neisseria meningitidis in the Xinjiang Uygur Autonomous Region, China ,2004-2023 | Page1，line1 |
| --- | --- | --- | --- |
| **Introduction** | | |  |
| Background/rationale | 2 | The Xinjiang Uygur Autonomous Region (Xinjiang) currently exhibits the high incidence rates of invasive meningococcal disease (IMD) in China. Current epidemiological data on meningococcal carriage rates among asymptomatic individuals remain sparse, with limited population-based studies systematically investigating the prevalence of Neisseria meningitidis colonization in the general population. | Page2，lines21-24 |
| Objectives | 3 | This study employs a dual-pronged epidemiological approach to systematically characterize the clinical-epidemiological profile of IMD in Xinjiang and quantify the nasopharyngeal carriage prevalence of N. meningitidis among asymptomatic populations, while identifying region-specific sociodemographic and behavioral determinants of carriage dynamics. | Page2，lines25-28 |
| **Methods** | | |  |
| Study design | 4 | Epidemiological characteristics were analyzed using descriptive epidemiological methods. N. meningitidis strains isolated from asymptomatic carriers underwent serogroup characterization via multiplex real-time polymerase chain reaction (Real-time PCR) targeting conserved capsular biosynthesis loci (serogroups A, B, C, W, X, and Y), with reaction conditions optimized per WHO standardized protocols for meningococcal molecular typing. The χ² test was used to compare the N. meningitidis carriage rates. A multivariable logistic regression model was used to analyze the risk factors associated with the carriage of N. meningitidis. | Page2，lines29-36 |
| Setting | 5 | During 2016, 2018, 2019, 2022, and 2023, a total of 3,075 participants were enrolled in cross-sectional surveys assessing *N.meningitidis* carriage prevalence in Xinjiang, stratified by northern and southern regions:Southern Xinjiang (n = 1,574): Aksu Prefecture: 250 participants、Hotan Prefecture: 422 participants、Kashgar Prefecture: 702 participants、Kizilsu Kirgiz Autonomous Prefecture (Kizilsu Prefecture): 200 participants；Northern Xinjiang (n = 1,501):Urumqi City: 278 participants、Karamay City: 212 participants、Turpan City: 242 participants、Tacheng Prefecture: 506 participants、Ili Kazak Autonomous Prefecture (Ili Prefecture): 263 participants | Page4，lines126-132 |
| Participants | 6 | Using random cluster sampling, the population was divided into six age groups, <3, 3~5, 6~10, 11~15, 16~20 and ≥21 years old, and throat swab specimens were collected. No less than 30 subjects were sampled in each age group, and 250 subjects were surveyed in each survey unit. The ratio of males to females was kept balanced. | Page4，lines132-135 |
| Variables | 7 | Age group(According to the national epidemic cerebrospinal meningitis surveillance Prescriptions are divided into six age groups), Gender, Years, Region, Vaccination history | Page7，lines238-259，Table 1 |
| Data sources/  measurement | 8* | Epidemiological Data Sources :The incidence data of IMD in the Xinjiang region from 2004 to 2023 in the Chinese Disease Prevention and Control Information System.  Data source of carrier rate: A cross-sectional survey of *Neisorrhea meningitidis* carrier rates was conducted in southern and northern Xinjiang in 2016, 2018, 2019, 2022 and 2023 | Page4，lines109-110，lines126-128 |

| Bias | 9 |  |  |
| --- | --- | --- | --- |
| Study size | 10 | 1. **Statistical Power**: We aimed for 80% power to detect a significant effect. 2. **Significance Level**: A significance level of 0.05 was set to limit the chance of false positives. 3. **Practical Considerations**: We also considered available resources and time constraints. | Page5，line 178 |
| Quantitative variables | 11 | The main type of data in this study is qualitative data.We grouped the data according to relevant surveillance protocols or demographic classifications for comparison. For example, age groups (e.g., <3, 3 to 5, 6 to 10, 11 to 15, 16 to 20 and ≥21 years old) were used to compare differences in carrier rates between different age groups. These groups were chosen to ensure meaningful comparisons and to be consistent with clinical significance. | Page4，line 133 |
| Statistical methods | 12 | Qualitative data are expressed as frequencies (n) and percentages (%). Comparisons of the positivity rates of *N. meningitidis* among different regions, sexes, age groups, years and immunization histories were performed using the χ2 test, with a p value <0.05 indicating a statistically significant difference. Regression Analysis: We conducted multiple regression analyses to examine the relationships between the dependent variable and several independent variables. This helped us identify significant predictors while controlling for potential confounders. | Page5，lines173-180 |
| **Results** | | |  |
| Participants | 13* | First, clearly describe the target population of the study, such as age, gender, region, occupation, etc. Ensure sample homogeneity and representativeness. It is emphasized that all participants participate voluntarily after fully understanding the purpose, methodology and potential risks of the study.Mention the informed consent signing process. If there are no personnel, analyze the possible impact of the reasons for not participating on the research results, such as sample bias and the universality of the conclusions. Suggest possible solutions or suggestions to mitigate these effects, such as expanding recruitment scope, improving recruitment strategies, etc. | Page7，lines135-136，168-171. |

| Descriptive data | 14* | | Age group, Gender, Years, Region, Vaccination history | Table 1 |
| --- | --- | --- | --- | --- |
| Outcome data | 15* | | A total of 3075 people were investigated, including 402 under 3 years old, 366 between 3 and 5 years old, 509 between 6 and 10 years old, 659 between 11 and 15 years old, 544 aged 16.20 years old and 595 aged ≥21 years old. There were 144 men and 1,609 women. 513 people were surveyed in 2016, 664 in 2018, 702 in 2019, 506 in 2022 and 690 in 2023. A total of 1,574 people were surveyed in southern Xinjiang and 1,501 in northern Xinjiang.A total of 3,075 throat swab specimens were collected from healthy people, and 411 N. meningitidis strains were detected, for an overall carriage rate of 13.37%. The most numerous group was group B, with 168 strains, more than one-third of the total number of strains. This was followed by 158 strains from the nongenogroup and 44 strains from group C, with proportions of 38.44% and 10.71%, respectively. | Page7，lines234-259 |
| Main results | 16 | | From 2004 to 2023, 1,100 cases of invasive meningococcal disease (IMD) were reported in Xinjiang, with the annual incidence rate fluctuating between 0.00/100,000 and 1.15/100,000 per year. The peak incidence occurred from February to May. The incidence was primarily concentrated in individuals under 20 years old (80.36%). A total of 3,075 pharyngeal swabs were investigated, of which 411 (13.37%) contained *N. meningitidis* strains. Among these, 168 (40.88%) strains belonged to group B, which was the most abundant group. Binomial multivariate logistic regression analyses revealed that age, sex, year, region, and vaccination history were risk factors affecting the *N. meningitidis* carriage rate (P < 0.05). | Page2，lines37-35 |
| Other analyses | 17 | |  |  |
| **Discussion** | | | |  |
| Key results | | 18 | Longitudinal surveillance (2004-2023) revealed a significant decline in IMD incidence across Xinjiang, transitioning from cyclical epidemic patterns to sustained hypoendemic transmission. Notwithstanding declining IMD incidence, N. meningitidis carriage persisted at elevated levels, with multilevel logistic regression identifying key determinants of regional heterogeneity. We recommend expanding the surveillance network for IMD and conducting annual population-based surveys of N. meningitidis carriage during early IMD outbreaks. This dual approach will enhance predictive capability for regional IMD trends, enabling timely adjustments to prevention and control strategies—ultimately strengthening evidence-based IMD management in Xinjiang. | Page11，lines424-432 |
| Limitations | | 19 | This study analyzed 20-year incidence data (2004–2023) of IMD cases in Xinjiang, derived from the China Disease Prevention and Control Information System. Data were aggregated from prefecture- and city-level CDCs and medical institutions across Xinjiang. A key limitation was that serogroup distribution data were not systematically collected by the reporting system, as case strains were not submitted to our laboratory for testing, and the system lacked predefined fields for serogroup information. Consequently, complete serogroup-specific data were unavailable, hampering a comprehensive analysis of the 20-year dataset and precluding a detailed assessment of serogroup trends over time. | Page10-11，lines409-421 |
| Interpretation | | 20 |  |  |
| Generalisability | | 21 | Currently, there are many studies on N. meningitidis carriage status around the world, and the carriage rate in healthy populations has changed considerably. In China, several provinces and cities have carried out surveys on N. meningitidis carriage rates. Therefore, this study studied the epidemic characteristics and the carrying status of the N. meningitidis in high-incidence areas of Invasive meningococcal disease, which could provide scientific basis for the prevention and control of Invasive meningococcal disease in our country. | Page3，lines91-97 |
| **Other information** | | | |  |
| Funding | | 22 | This work received a grant from the Tianshan Yingcai Medical and Health Talent Cultivation Program (NO. TSYC202301B097) and Xinjiang Natural Population Cohort Construction and Active Health Innovation Team, China (NO. 2022TSYCTD0013). The funders had no role in study design, data collection and analysis, decision to publish, or preparation of the manuscript. | Page11，lines436-440 |

*Give information separately for exposed and unexposed groups.

**Note:** An Explanation and Elaboration article discusses each checklist item and gives methodological background and published examples of transparent reporting. The STROBE checklist is best used in conjunction with this article (freely available on the Websites of PLoS Medicine at <http://www.plosmedicine.org/>, Annals of Internal Medicine at

<http://www.annals.org/>, and Epidemiology at <http://www.epidem.com/>). Information on the STROBE Initiative is available at www.strobe-statement.org.
