# Supplementary material for: Epidemiological characteristics of invasive meningococcal disease and carriage prevalence of Neisseria meningitidis in the Xinjiang Uygur Autonomous Region, China, 2004–2023: a retrospective study
Source: PeerJ. 2025 Jul 29;13:e19772. doi: 10.7717/peerj.19772 (PMC12315827; doi:10.7717/peerj.19772)
Supplement: Supplemental Information 3 [file peerj-13-19772-s003.doc]

****Table 1: Specimen Collection Registration Form for Carrier Survey in Healthy Populations****

| **number** | **name** | **ID card number** | **Gender (1. Male; 2. Female)** | **Date of birth (or age)** | **nationality** | **careers** | **Address (mode of residence: 1 Diaspora 2 Congregation)** | **Household registration (1 local 2 mobile)** | **educational attainment** | **1 week of respiratory disease (1 yes; 2 no), indicating type of disease** | **Any antibiotics taken during the week (1 yes; 2 no 3 unknown) Indicate type of medication** | **vaccinations** | | | | | **note** |
| --- | --- | --- | --- | --- | --- | --- | --- | --- | --- | --- | --- | --- | --- | --- | --- | --- | --- |
|  |  |  |  |  |  |  |  |  |  |  |  | **Nm** | | | **Bp** | |  |
|  |  |  |  |  |  |  |  |  |  |  |  | **Type of vaccination (1. Group A; 2. Group A+C; 3. Group A with Group A+C;; 4. Unknown; 5. Unvaccinated)** | **Number of inoculations** | **Date of last vaccination (year/month/day)** | **1 dose, 2 doses, 3 doses, enhanced** | **Date of last vaccination (year/month/day)** |  |
|  |  |  |  |  |  |  |  |  |  |  |  |  |  |  |  |  |  |
|  |  |  |  |  |  |  |  |  |  |  |  |  |  |  |  |  |  |
|  |  |  |  |  |  |  |  |  |  |  |  |  |  |  |  |  |  |
|  |  |  |  |  |  |  |  |  |  |  |  |  |  |  |  |  |  |
|  |  |  |  |  |  |  |  |  |  |  |  |  |  |  |  |  |  |
|  |  |  |  |  |  |  |  |  |  |  |  |  |  |  |  |  |  |
|  |  |  |  |  |  |  |  |  |  |  |  |  |  |  |  |  |  |
|  |  |  |  |  |  |  |  |  |  |  |  |  |  |  |  |  |  |
| nationality: 1. Han , 2 Uyghur, 3. Kazakh, 4. Hui, 5. Other (please indicate specific ethnicity). | | | | | | | | | | | | | | | | | |
| careers: 1. Diaspora children, 2. Childcare children, 3. Students, 4. Teachers, 5. Doctors, 6. Farmers, 7. Individuals, 8. Others | | | | | | | | | | | | | | | | | |
| educational attainment: 1. Elementary school and below, 2. Middle school, 3. High school or junior college, 4. College, 5. Bachelor's degree and above | | | | | | | | | | | | | | | | | |

Specimen collector: inspection unit： sender： Date of survey：
