# Supplementary material for: Epidemiological characteristics of invasive meningococcal disease and carriage prevalence of Neisseria meningitidis in the Xinjiang Uygur Autonomous Region, China, 2004–2023: a retrospective study
Source: PeerJ. 2025 Jul 29;13:e19772. doi: 10.7717/peerj.19772 (PMC12315827; doi:10.7717/peerj.19772)
Supplement: Supplemental Information 4 [file peerj-13-19772-s004.doc]

**表1 年 市健康人群带菌调查标本采集登记表**

| **编号** | **姓名** | **身份证号** | **性别（1.男；2.女）** | **出生年月(或岁)** | **民族** | **职业** | **住址（居住方式：1 散居 2 聚居）** | **户籍（1本地 2流动）** | **文化程度** | **1周有无呼吸系统疾病（1 有；2 无），注明疾病种类** | **一周内有无服用抗生素（1 有；2 无 3 不详）注明药物种类** | **疫苗接种** | | | | | **备注** |
| --- | --- | --- | --- | --- | --- | --- | --- | --- | --- | --- | --- | --- | --- | --- | --- | --- | --- |
|  |  |  |  |  |  |  |  |  |  |  |  | **流脑** | | | **百日咳** | |  |
|  |  |  |  |  |  |  |  |  |  |  |  | **接种疫苗种类(1.A群;2.A+C群;3.A群与A+C群;；4.不详；5.未种)** | **接种次数** | **最后一次接种日期(年/月/日)** | **1剂次、2剂次、3剂次、加强** | **最后一次接种日期(年/月/日)** |  |
|  |  |  |  |  |  |  |  |  |  |  |  |  |  |  |  |  |  |
|  |  |  |  |  |  |  |  |  |  |  |  |  |  |  |  |  |  |
|  |  |  |  |  |  |  |  |  |  |  |  |  |  |  |  |  |  |
|  |  |  |  |  |  |  |  |  |  |  |  |  |  |  |  |  |  |
|  |  |  |  |  |  |  |  |  |  |  |  |  |  |  |  |  |  |
|  |  |  |  |  |  |  |  |  |  |  |  |  |  |  |  |  |  |
|  |  |  |  |  |  |  |  |  |  |  |  |  |  |  |  |  |  |
|  |  |  |  |  |  |  |  |  |  |  |  |  |  |  |  |  |  |
|  |  |  |  |  |  |  |  |  |  |  |  |  |  |  |  |  |  |
|  |  |  |  |  |  |  |  |  |  |  |  |  |  |  |  |  |  |
| 民族：1.汉族，2维吾尔族，3.哈萨克族，4.回族，5.其他（请注明具体民族）。 | | | | | | | | | | | | | | | | | |
| 职业：1.散居儿童，2.托幼儿童，3.学生，4.教师，5.医生，6.农民，7.个体，8.其他 | | | | | | | | | | | | | | | | | |
| 文化程度：1.小学及以下， 2.初中， 3.高中或中专， 4.大专， 5.本科及以上 | | | | | | | | | | | | | | | | | |

标本采集人： 送检单位： 送检人： 调查日期：
